# Presence and distribution of progerin in HGPS cells is ameliorated by drugs that impact on the mevalonate and mTOR pathways

**DOI:** 10.1007/s10522-019-09807-4

**Published:** 2019-04-30

**Authors:** Craig S. Clements, Mehmet U. Bikkul, Wendy Ofosu, Christopher Eskiw, David Tree, Evgeny Makarov, Ian R. Kill, Joanna M. Bridger

**Affiliations:** 1 Progeria Research Team, Ageing Studies Theme, Institute for Environment, Health and Societies, Brunel University London, Kingston Lane, Uxbridge, UB8 3PH UK; 2 Department of Biomedical Sciences, University of Westminster, 115 New Cavendish Street, London, W1W 6UW UK; 3 Food and Bioproduct Sciences, College of Agriculture and Bioresources, University of Saskatchewan, 51 Campus Drive, Saskatoon, SK S7B 5A8 Canada; 4 Genome Engineering and Maintenance Network, Ageing Studies Theme, Institute of Environment, Health and Societies, Brunel University London, Kingston Lane, Uxbridge, UB8 3PH UK

**Keywords:** Nuclear lamins, Hutchinson–Gilford progeria syndrome, Farnesyl transferase inhibitors, Rapamycin, N-acetyl cysteine, Pravastatin, Zoledronic acid, Progerin, Internal lamin foci

## Abstract

Hutchinson–Gilford progeria syndrome (HGPS) is a rare, premature ageing syndrome in children. HGPS is normally caused by a mutation in the *LMNA* gene, encoding nuclear lamin A. The classical mutation in HGPS leads to the production of a toxic truncated version of lamin A, progerin, which retains a farnesyl group. Farnesyltransferase inhibitors (FTI), pravastatin and zoledronic acid have been used in clinical trials to target the mevalonate pathway in HGPS patients to inhibit farnesylation of progerin, in order to reduce its toxicity. Some other compounds that have been suggested as treatments include rapamycin, IGF1 and *N*-acetyl cysteine (NAC). We have analysed the distribution of prelamin A, lamin A, lamin A/C, progerin, lamin B1 and B2 in nuclei of HGPS cells before and after treatments with these drugs, an FTI and a geranylgeranyltransferase inhibitor (GGTI) and FTI with pravastatin and zoledronic acid in combination. Confirming other studies prelamin A, lamin A, progerin and lamin B2 staining was different between control and HGPS fibroblasts. The drugs that reduced progerin staining were FTI, pravastatin, zoledronic acid and rapamycin. However, drugs affecting the mevalonate pathway increased prelamin A, with only FTI reducing internal prelamin A foci. The distribution of lamin A in HGPS cells was improved with treatments of FTI, pravastatin and FTI + GGTI. All treatments reduced the number of cells displaying internal speckles of lamin A/C and lamin B2. Drugs targeting the mevalonate pathway worked best for progerin reduction, with zoledronic acid removing internal progerin speckles. Rapamycin and NAC, which impact on the MTOR pathway, both reduced both pools of progerin without increasing prelamin A in HGPS cell nuclei.

**Electronic supplementary material:**

The online version of this article (10.1007/s10522-019-09807-4) contains supplementary material, which is available to authorized users.

## Introduction

The nuclear lamina underlying the inner nuclear membrane, consists of separate mesh-works of class V intermediate filament proteins known as nuclear lamins (Aebi et al. 1986; de Leeuw et al. 2018). The importance of a correctly structured lamina is borne out by the comprehensive range of cellular functions that it is involved with (Bridger et al. 2014). This range includes structural support (Tokes and Clawson 1989; Schäpe et al. 2009), transcriptional regulation (Andrés and González 2009; Dechat et al. 2008), positioning of nuclear pores (Al-Haboubi et al. 2011), DNA replication (Hutchison et al. 1994), nuclear envelope breakdown and reformation during mitosis (Gerace and Blobel 1980) and organisation of the genome (Andrés and González 2009; Meaburn et al. 2007), through lamina associated domains (LADs) (Guelen et al. 2008). There are two types of lamins in animal cells, A-type lamins which are well expressed in differentiated cells following gastrulation (Broers et al. 1997), and B-type lamins. Due to alternative splicing, the lamin A gene generates several isoforms, including lamin A, C, C2 and lamin AΔ10 (Cau et al. 2014; Luo et al. 2014). On the other hand, the two B-type lamins, B1 and B2, are expressed from genes located on different chromosomes. Lamin B2 in particular is present in the vast majority of cell types, with the exception of hepatocytes, unlike lamin B1 which does not appear to be expressed in muscle or connective tissue in mammalian cells (Broers et al. 1997).

A-type and B-type lamins are initially expressed as precursors in the cytosol and referred to as prelamins (Beck et al. 1990). Although sharing similarities in structure, analysis on *Xenopus* oocytes and mouse cells demonstrated the two lamin sub-types are in fact two classes of proteins which assemble independently (Goldberg et al. 2008; Sullivan et al. 1999), and in order to carry out their function a number of post-translational modifications must be first completed.

All lamins possess an N-terminal “head” domain and four central alpha-helices interspersed by linker regions. Through this complex, denoted “rod domain”, lamins interact with one another and assemble into the nuclear envelope. The terminal portion of the protein is defined by a C-terminus containing a -CAAX box motif, which is crucial to the lamin’s functionality (Holtz et al. 1989; Vorburger et al. 1989; Reddy and Comai 2012). Nuclear lamins undergo, post-translational modifications that involve the farnesylation of the cysteine belonging to the -CAAX terminal box by the farnesyltransferase enzyme (Sinensky et al. 1994). This enables incorporation of the proteins into the endoplasmic reticulum (Bergo et al. 2002). This process is followed by a proteolytic cleavage after the cysteine residue and a successive carboxymethylation of the terminal part of the compound, operated by the isoprenyl-cysteine-carboxy-methyltransferase (ICMT) enzyme (Beck et al. 1990). B-type lamins remain farnesylated and localise to the inner nuclear envelope by lateral diffusion through the lipid bilayer (Holmer and Worman 2001). The mature version of lamin A is produced by a second proteolytic cleavage catalysed by the Zinc Metallo-Protease STE24 (ZMPSTE24) (Freije et al. 1999), causing the deletion of 15 amino acids, including the modified cysteine where the farnesylation occurs. This allows the soluble protein to be imported into nuclei through the nuclear pore complexes (Cau et al. 2014).

Hutchinson–Gilford Progeria Syndrome (HGPS) is a fatal premature ageing syndrome in children with an incidence rate of 1 in 8 million live births (Pollex and Hegele 2004; Gonzalo et al. 2017). Affected individuals usually die in their teens (Worman et al. 2010; Gonzalo et al. 2017). The typical characteristics presented with HGPS include short stature, full-body alopecia, decreased joint mobility, lack of subcutaneous fat and muscle, osteolysis, micrognathia, and coxa valga (de Paula Rodrigues et al. 2002; Merideth et al. 2008). While there are a number of mutations in different genes that can cause HGPS the most common mutation associated with “classical” HGPS is caused by a de novo mutation in the lamin A (LMNA) gene (De Sandre-Giovannoli, et al. 2003; Eriksson, et al. 2003). Even though it is a silent mutation found at codon 608 (G608G, GGC > GGT) of the gene, it leads to the increased usage of a cryptic splice site. This results in the deletion of 150 nucleotides from exon 11 of the lamin A transcript (Eriksson, et al. 2003) that includes the ZMPSTE24 cleavage site for processing the immature lamin A (Freije et al. 1999; Pendas et al. 2002). The aberrant splicing leads to the formation and accumulation of a dominant negative pre-lamin protein (Eriksson et al. 2003) which remains anchored to the nuclear membrane, a property associated with B-type lamins and not A-type. The failure of the mutant lamin A protein to be cleaved results in a permanently farnesylated toxic protein, termed ‘*progerin*’ (Yang et al. 2005), that accumulates in the nuclear membrane. This mechanism is thought to be the underlying cause of premature aging in HGPS (Bridger and Kill 2004; Columbaro et al. 2005). The nuclei of HGPS fibroblasts develop a distorted shape, exhibiting blebs, herniations and lobulations, as well as the formation of micronuclei (Bridger and Kill 2004; Goldman et al. 2004; Paradisi et al. 2005). Progerin has also been revealed in normal aged cells both in vivo and in vitro, highlighting one of the main correlations between HGPS and normal ageing (Scaffidi and Misteli 2006). Indeed, various forms of farnesylated lamin A are thought to be involved in the ageing process (Reddy and Comai 2012; Cao et al. 2011; McClintock et al. 2007; Bonello-Palot et al. 2014).

Therapeutic strategies for treating HGPS involve blocking the farnesylation of progerin (Gordon et al. 2014, Varela et al. 2008) or the activation of pathways that facilitate the removal/degradation of progerin (Cao et al. 2011; Mendelsohn and Larrick 2011). One option is to inhibit the synthesis of farnesyl precursors through disruption of major biochemical pathways responsible for its production, the mevalonate pathway. Several drugs exist that inhibit this pathway at several points blocking the farnesylation of progerin. By inhibiting the farnesylation of progerin it is believed to reduce the toxicity of the protein as it would be unable to incorporate into and accumulate within the nuclear envelope. FTIs are such drugs that act on the mevalonate pathway and are being employed as anti-cancer therapeutics since these drugs inhibit the farnesylation of the ras protein, commonly mutated in cancers (Sun et al. 1995). While studies have shown that FTIs can restore nuclear shape initially in mouse models of progeria (Yang et al. 2005), in human HGPS fibroblasts (Glynn and Glover 2005) and in mesenchymal stem cells (MSC) derived from HGPS iPS cells (Blondel et al. 2014), deleterious effects of FTI treatment have been noted. FTI treatment can lead to the formation of abnormal donut-shaped nuclei (Verstraeten et al. 2011) and can redistribute normal A-type lamins away from the inner nuclear envelope (Wang et al. 2012). Furthermore, prelamin A has been shown to be over-expressed and accumulate in MSC derived from HGPS iPS cells (Blondel et al. 2014). Pravastatin and zoledronic acid (an amino-bisphosphonate), which inhibit the HMG-CoA reductase and FPP synthetase enzymes of the mevalonate pathway respectively, have been also been used as treatments for HGPS since statins and amino-bisphosphonates have been demonstrated to improve longevity in ZMPSTE24 deficient mouse models (Varela et al. 2008). The effectiveness of FTI alone and in a combination treatment with pravastatin and zoledronic acid in restoring nuclear shape has been previously shown in in vivo mouse models (Wang et al. 2010).

Three clinical trials for HGPS sufferers were active this decade. Firstly, a trial using lonafarnib (an FTI) alone for 2 years was reported to have benefits for the children in bone structure, vascular stiffness and hearing (Gordon et al. 2014) and survival (Gordon et al. 2018). A subsequent trial was a three drug combination of lonafarnib, pravastatin and zoledronic acid based in the United States at Children’s Hospital Boston (Clinical trial ID: NCT00879034). This trial has reported that using the two additional drugs have resulted in an increase in bone mineral density (Gordon et al. 2016). A trial using a two drug combination of pravastatin and zoledronic acid based in France at the Assistance Publique Hopitaux De Marseille (Clinical trial ID: NCT00731016) for HGPS patients has concluded. The combination of a bisphosphonate and pravastatin have shown improvements in skin ageing external markers i.e. wrinkles in one study (Cantecor et al. 2013). We have found previously that FTIs alone, and in combination with pravastatin and zoledronic acid place chromosomes back into their correct locations in interphase nuclei (Bikkul et al. 2018, Bridger et al. 2014) and that an active mechanism for their specific relocation is also restored with FTI (Mehta et al. 2011).

FTI inhibition through farnesylation of progerin and prelamin A can be made more efficient through the additional use of geranylgeranyltransferase inhibitors (GGTIs) (Varela et al. 2008). Geranylgeranyltransferase adds a lipophilic geranylgeranyl moiety to proteins than contain CAAX motifs (Yoshida et al. 1991). Other drugs have also been presented as having the potential to treat progeria patients and are going into trial for progeria, such as the rapalogue of rapamycin, everolimus (Clinical Trial ID NCT02579044). Rapamycin, a mechanistic target of rapamycin (mTOR) inhibitor, has been shown to decrease insoluble progerin aggregates within nuclei of HGPS fibroblasts through autophagic degradation (Cao et al. 2011, Cenni et al. 2011) and have an positive effect on MSC derived from HGPS iPS cells by reducing the number of progerin expressing cells (Blondel et al. 2014). Further, rapamycin has been demonstrated to reduce nuclear blebbing in HGPS fibroblast cells (Driscoll et al. 2012) and improve nuclear shape in MSC derived from HGPS iPS cells in a quarter of the population (Blondel et al. 2014). More clinically relevant rapalogues such as temsirolimus and everolimus generate a range of improvements in HGPS cells; with temsirolimus reducing progerin levels, increasing proliferation, correcting mis-shaped nuclei and improving the fraction of DNA damage foci (Gabriel et al. 2016). ZMPSTE24 (−/−) mice have been shown to be deficient in insulin-like growth factor 1 (IGF-1) and exhibit elevated levels of growth hormone (GH). Administering recombinant IGF-1 protein to the mice restores the GH/IGF-1 balance and increases their longevity (Marino et al. 2010). HGPS fibroblasts have been shown to have DNA repair defects, exhibiting elevated levels of DNA double stand breaks (DSB) (Liu et al. 2005). This damage is thought to be due to the generation of reactive oxygen species (ROS) (Pereira et al. 2008). *N*-acetyl-l-cysteine (NAC) is a ROS scavenger that has been shown to reduce the levels of ROS-induced DSBs in HGPS fibroblasts (Richards et al. 2011). However, NAC is also an activator of the mTOR pathway (Chen et al. 2014).

While extensive studies using proposed drugs for HGPS treatment have been conducted using mouse models, relatively few studies have used primary cells to investigate their effect on the distribution of nuclear proteins, such as lamins. The effect of drugs that do not target the mevalonate pathway on lamins in HGPS fibroblasts is largely unknown. To address this current gap in knowledge, the purpose of this study was to test whether or not the proposed drugs and combinations of drugs used in clinical trials can improve the distribution of nuclear lamins in HGPS fibroblasts. We have found using careful analysis of indirect immunofluorescent staining patterns, that drugs which prevent farnesylation of progerin through the mevalonate pathway and drugs the activate the mTOR pathway restore the nuclear distribution and complement of nuclear lamins back to a normal level.

## Materials and methods

### Cell culture

The AG01972 primary HGPS cell line was obtained from Coriell Cell Repositories (New Jersey, USA). The 2DD normal dermal fibroblast cell line was used as described previously (Bridger et al. 1993). AG01972 cells were cultured in T75 tissue culture treated flasks (Fisher, UK) containing Dulbecco’s Minimum Essential Media + Glutamax (Invitrogen, UK), supplemented with 15% foetal calf serum and 1% penicillin/streptomycin (Invitrogen) in an humidified, 37 °C, 5% CO_2_/95% air incubator. The cells were passaged twice weekly. Cells were plated at a density of 2 × 10^5^ into 9 cm dishes containing 13 mm diameter glass coverslips.

### Antibodies and drug treatments

The antibodies used in this investigation were anti-prelamin A (SC-6214, goat polyclonal IgG, Santa Cruz, USA) at a 1:50 dilution, anti-lamin A (AB8980, mouse monoclonal IgG3, Abcam, UK) at a 1:50 dilution, anti-lamin A/C (NCL-LAM-A/C, mouse monoclonal IgG2B, Leica Biosystems, Germany) at a 1:100 dilution, anti-progerin (ALX-804-662-R200, mouse monoclonal IgG1, Enzo Life Sciences, USA) at a 1:50 dilution, anti-lamin B1 (AB8982, mouse monoclonal IgG1, Abcam, UK) at a 1:100 dilution and anti-lamin B2 (AB8983, mouse polyclonal IgG1, Abcam, UK) at a 1:100 dilution. The secondary antibodies used were FITC conjugated donkey anti-mouse IgG (715-095-150, Jackson Immunosearch, USA) and FITC conjugated donkey anti-goat IgG (705-095-147, Jackson Immunosearch, USA) at 1:50 dilution. The drugs used were FTI-277, pravastatin, zoledronic acid, rapamycin, insulin-like growth factor 1, *N*-acetyl-l-cysteine and GGTI-2133, were obtained from Sigma Aldrich, UK. Drugs were added to the media and incubated for fixed periods of time. The final concentration and duration of drug treatments followed were extracted from previous studies and as such relevant to whole organisms or other in vitro studies: FTI-277 and GGTI-2133—2.5 µM for 48 h (Kieran et al. 2007; Mehta et al. 2010), pravastatin and zoledronic acid—1 µM for 24 h (Varela et al. 2008), *N*-acetyl-l-cysteine—20 µM for 1 h (Richards et al. 2011), IGF1—50 ng ml^−1^ for 24 h (Marino et al. 2010) and rapamycin—10 nM for 24 h (Cao et al. 2011).

### Indirect immunofluorescence

Cells grown on coverslips were washed three times in PBS and then fixed with ice-cold methanol:acetone (1:1). Cells were then further washed three times in PBS. Primary antibodies were applied to the coverslip for 1 h, and then washed in a PBS row. The secondary antibodies are then applied for 1 h, followed by further washing in a PBS row. The coverslips were then mounted onto glass slides with Vectashield Mounting Medium containing DAPI (Vector Labs). Nuclei were imaged using an Olympus BX-41 fluorescence microscope (Olympus Corporation, Japan) with an UPlanFl ×100/1.30 objective lens (Olympus Corporation, Japan). Images were captured with Viewpoint GS digital camera and SmartCapture 3 software for Apple Mac for the same length of exposure (both Digital Scientific UK).

### Data analysis

The images of antibody stained nuclei were manually scored for criteria. The number of nuclei exhibiting a given pattern was expressed as a percentage of the total number of nuclei scored in that analysis. Values are expressed as averages ± SEM, and n represents the number of experiments analysed. Individual treatments were compared using two-way ANOVAs with Tukey’s post hoc multiple comparison tests, and significance was taken as P ≤ 0.05. The level of significance is indicated as: * P ≤ 0.05, ** P ≤ 0.01, *** P ≤ 0.001 and **** P ≤ 0.0001. Analysis was performed using Graphpad Prism 6.0 for Windows package.

## Results

Nuclear lamins are found at the nuclear periphery in a rim subjacent to the inner nuclear membrane, but also as internal speckle structures deep within nuclei. This is true not only for lamin A (Bridger et al. 1993; Goldman et al. 1992), but also prelamin A (Sasseville and Raymond 1995), and even B-type lamins (Moir et al. 1994) can be observed located within internal foci. In this study, we have analysed nuclear lamin presence and distribution before and after a panel of single and combinatorial drug treatments in HGPS primary fibroblasts. We defined a series of patterns for the staining of both A-type and B-type lamins including prelamin A and progerin to not only include the nuclear periphery but also to take into consideration internal lamin foci. Thus, these patterns are described as four distinct sub-categories: (1) rim only, (2) rim with internal speckles, (3) internal speckles only and, (4) negative. For prelamin A the rim staining has been sub-categorised into strong and weak to differentiate between effects of different drugs. Research looking at the distribution of nuclear lamins in HGPS fibroblasts, including B-type lamins, has been previously performed (Adam et al. 2013), however only FTIs and GGTIs were studied. In this study, we have used a much wider range of actual and proposed drug treatments for HGPS to reveal their effects on the lamin distribution in fibroblast nuclei.

### Prelamin A distribution

**Fig. 1:**
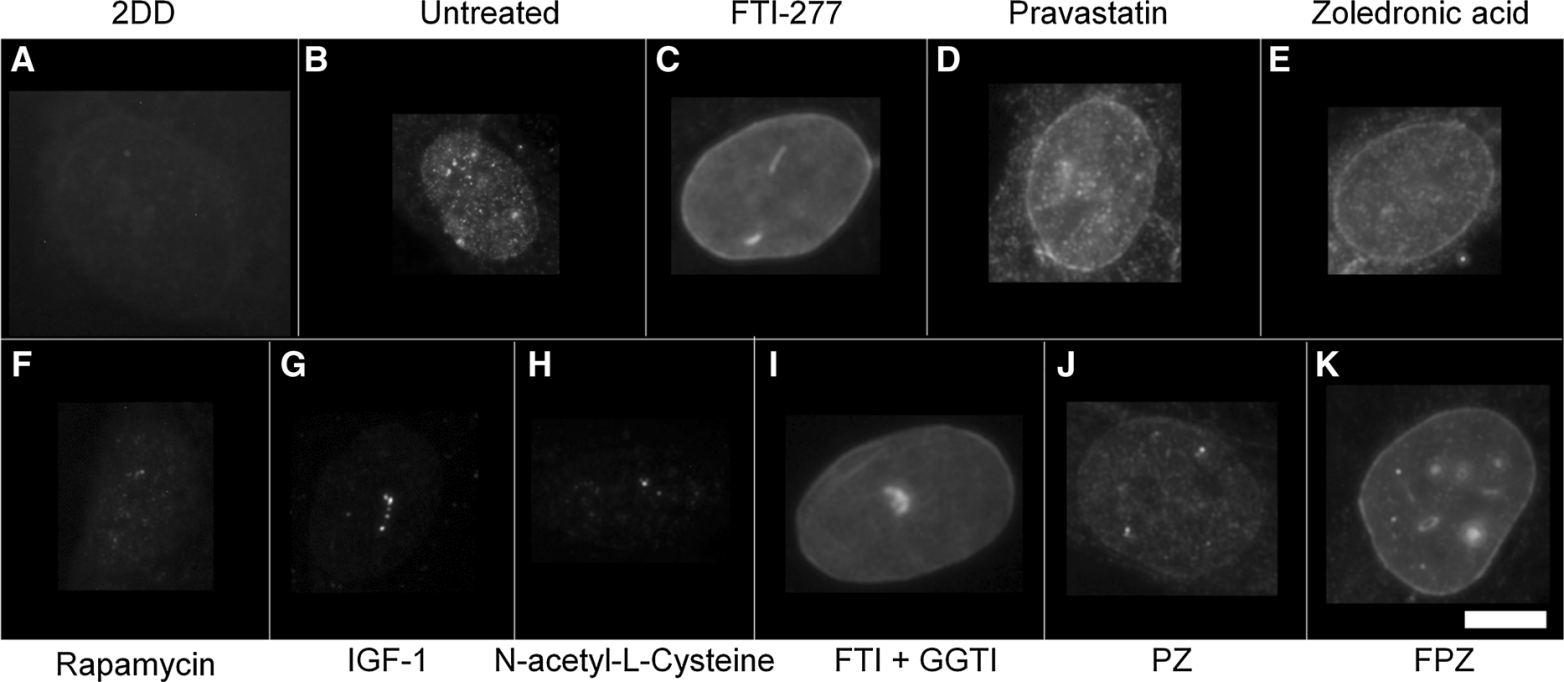
Representative images of prelamin A staining of HGPS fibroblast nuclei. Representative images of HGPS nuclei fixed with methanol:acetone and stained using a prelamin A primary antibody and a FITC-conjugated secondary antibody. Images captured at ×100 magnification using an Olympus BX-41 fluorescence microscope with an UPlanFl ×100/1.30 objective lens (Olympus Corporation, Japan). Images were captured with Viewpoint GS digital camera and SmartCapture 3 software for Apple Mac (both Digital Scientific UK). The treatments are shown as **a** untreated 2DD **b** untreated AG01972, **c** AG01972 with FTI-277 (2.5 µM for 24 h), **d** AG01972 with pravastatin (1 µM for 24 h), **e** AG01972 with zoledronic acid (1 µM for 24 h), **f** AG01972 with rapamycin (10 nM for 24 h), **g** AG01972 with insulin-like growth factor 1 (50.0 ng ml-1 for 24 h), **h** AG01972 with *N*-acetyl-l-cysteine (20 µM for 1 h), **i** AG01972 with FTI-277 and GGTI-2133 (both 2.5 µM for 24 h), **j** AG01972 with pravastatin and zoledronic acid (both 1 µM for 24 h) and **k** AG01972 with FTI-277 (2.5 µM for 24 h), pravastatin and zoledronic acid (both 1 µM for 24 h). Scale bar = 10 µM

**Fig. 2:**
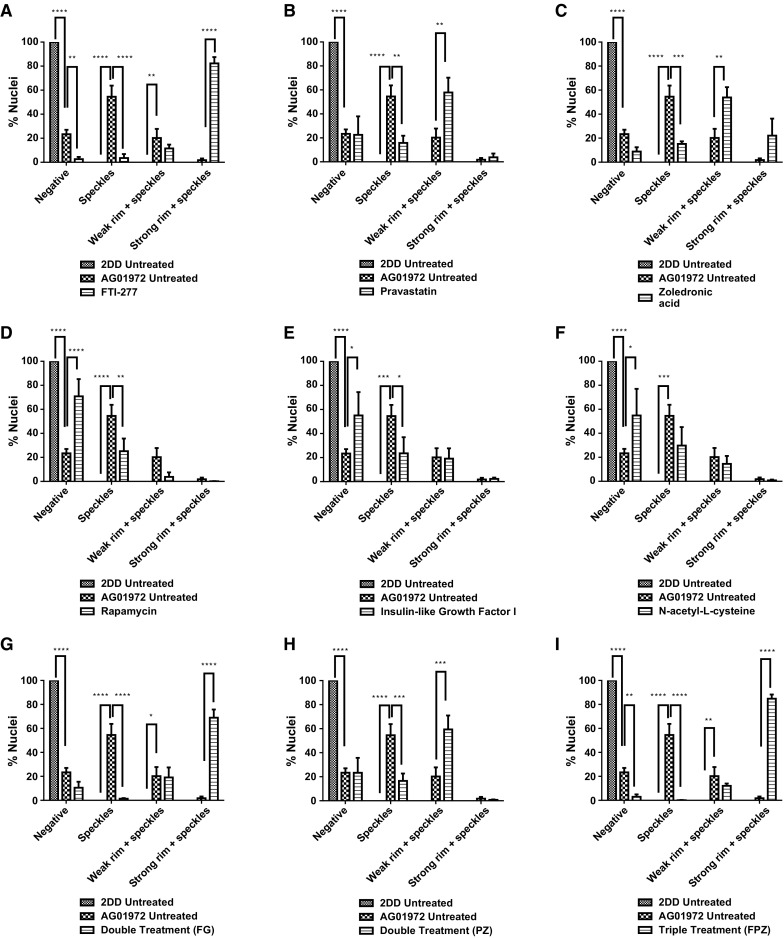
Scoring of prelamin A stained AG01972 fibroblasts after drug treatment. 2DD and AG01972 nuclei fixed with methanol:acetone and stained using prelamin A primary antibodies and FITC-conjugated secondary antibodies were scored by eye using an Olympus BX-41 fluorescence microscope with an UPlanFl ×100/1.30 objective lens (Olympus Corporation, Japan). The scoring patterns used for the analysis were negative, speckles, aggregates, weak rim and speckles, and strong rim and speckles. Scoring results for the treatments are shown as **a** FTI-277 (2.5 µM for 24 h), **b** pravastatin (1 µM for 24 h), **c** zoledronic acid (1 µM for 24 h), **d** rapamycin (10 nM for 24 h), **e** insulin-like growth factor 1 (50.0 ng ml^−1^ for 24 h), **f***N*-acetyl-l-cysteine (20 µM for 1 h), **g** FTI-277 and GGTI-2133 (both 2.5 µM for 24 h), **h** pravastatin and zoledronic acid (both 1 µM for 24 h) and **i** FTI-277 (2.5 µM for 24 h), pravastatin and zoledronic acid (both 1 µM for 24 h). Error bars are Standard error of the mean (SEM)

To determine the impact of single and combinatorial treatments of drugs on pre-lamin A distribution we employed control and HGPS primary fibroblasts and indirect immunofluorescence. In control fibroblasts it was difficult to reveal any prelamin A staining since it is processed rapidly and thus most of the cells appear negative (Figs. 1, 2, Supplementary Table 1). However, the untreated HGPS fibroblasts exhibit a majority of cells with prelamin A internal speckles (55%) and a weak rim staining in addition to internal speckles (20%). These data suggest that in HGPS fibroblasts, prelamin A is not only accumulating at foci within the nucleoplasm but also additionally at the nuclear rim. Following FTI-277 treatment (Fig. 2a), there is a significant reduction (55% > 3.5%) in the proportion of HGPS cells displaying only internal speckles of prelamin A compared to the untreated HGPS fibroblast cells. Conversely, there is a significant increase in the proportion of cells displaying a strong nuclear rim staining with internal foci (82%) compared to the untreated HGPS fibroblast (1.8%). These data are in agreement with similar analyses suggesting that prelamin A staining intensifies at the nuclear rim in HGPS fibroblasts in response to FTI treatment (Adam et al. 2013). Following pravastatin treatment (Fig. 2b), there is a significant reduction in the fraction of cells displaying internal speckles only (13%) compared to the untreated HGPS fibroblasts (43%). There is also a significant increase in the fraction of HGPS cells displaying a weak rim and internal speckles (58%) compared to the untreated HGPS fibroblast cells (20%) indicating that prelamin A is again re-localising to the nuclear rim. Following zoledronic acid treatment (Fig. 2c), there is a significant reduction in the fraction of cells displaying intranuclear speckles (13%) compared to the untreated HGPS fibroblast cells (43%). However, in a similar result as that revealed with pravastatin treatment, there is a significant increase in the proportion of weak rim and speckle staining cells (52%) compared to the untreated HGPS fibroblast cells (20%). Following rapamycin treatment (Fig. 2d), there is a significant reduction in the fraction of cells displaying internal speckles (17%) compared to the untreated HGPS fibroblasts (43%). There is also highly significant increase in the fraction of cells with negative staining for prelamin A (71%) compared to the untreated HGPS fibroblast cells (23%), bringing the overall results for rapamycin treatment with respect to prelamin A in line with those of the control fibroblasts.

Following IGF-1 treatment (Fig. 2e), there is also significant reduction in the proportion of cells revealing internal prelamin A speckles (7%) compared to the untreated HGPS fibroblast cells (43%). Conversely, there was also a significant increase in the proportion of negative staining cells (55%) compared to the untreated HGPS fibroblast cells (23%). Similarly to rapamycin, the results indicate a pattern more consistent to that of the control cells. Following NAC treatment (Fig. 2f), there was again a significant reduction in the proportion of cells displaying internal speckles (15%) compared to the untreated HGPS fibroblast cells (43%). There was also a significant increase in the proportion of negative staining (55%) compared to the untreated HGPS fibroblast cells (23%). Following combinatorial treatment with FTI-277 and GGTI-2133 (Fig. 2g), there was a significant reduction in the proportion of HGPS cells displaying internal speckles (1%). There was also a significant increase in the proportion of strong rim and speckles (69%) compared to the untreated HGPS fibroblast cells (1.8%). This result is very similar to that of FTI-277 treatment alone, where there is an accumulation of prelamin A at the nuclear rim following treatment. There was a significant reduction in the proportion of cells with speckles following combinatorial treatment of pravastatin and zoledronic acid (4%) (Fig. 2 h) compared to the untreated HGPS fibroblasts. Contrary to this, there was a significant increase in the proportion of weak rim and speckles (59%) compared to the untreated HGPS fibroblast controls (21%). The result is very similar to that of pravastatin and zoledronic acid treatments alone. Following the combinatorial treatment of FTI-277, pravastatin and zoledronic acid (Fig. 2i), there was a significant reduction in the fraction of HGPS cells displaying internal speckles (0.1%) and negative staining (3%) compared to the untreated HGPS fibroblast cells. However, there is also a significant increase in the proportion of strong rim and speckles (85%) compared to the untreated HGPS fibroblast cells. This result resembles that of FTI-277 alone, and combination treatment of FTI-277 and GGTI-2133. In general, it was further noted that the addition of FTIs in any combination led to a characteristic dull prelamin A stain across the nucleoplasm (Fig. 1c, i, k). This was also present, but to a lesser extent, when using pravastatin and zoledronic acid (Fig. 1d, e, j). Taken together the data generated by these analyses reveal that Rapamycin, IGF1 and NAC would be the drugs that bring the HGPS cells closer to control cells by increasing the proportion of prelamin A negative cells. The drugs that inhibit the farnesylation create HGPS cells with increased staining at the nuclear envelope and with internal foci.

### Lamin A distribution

**Fig. 3:**
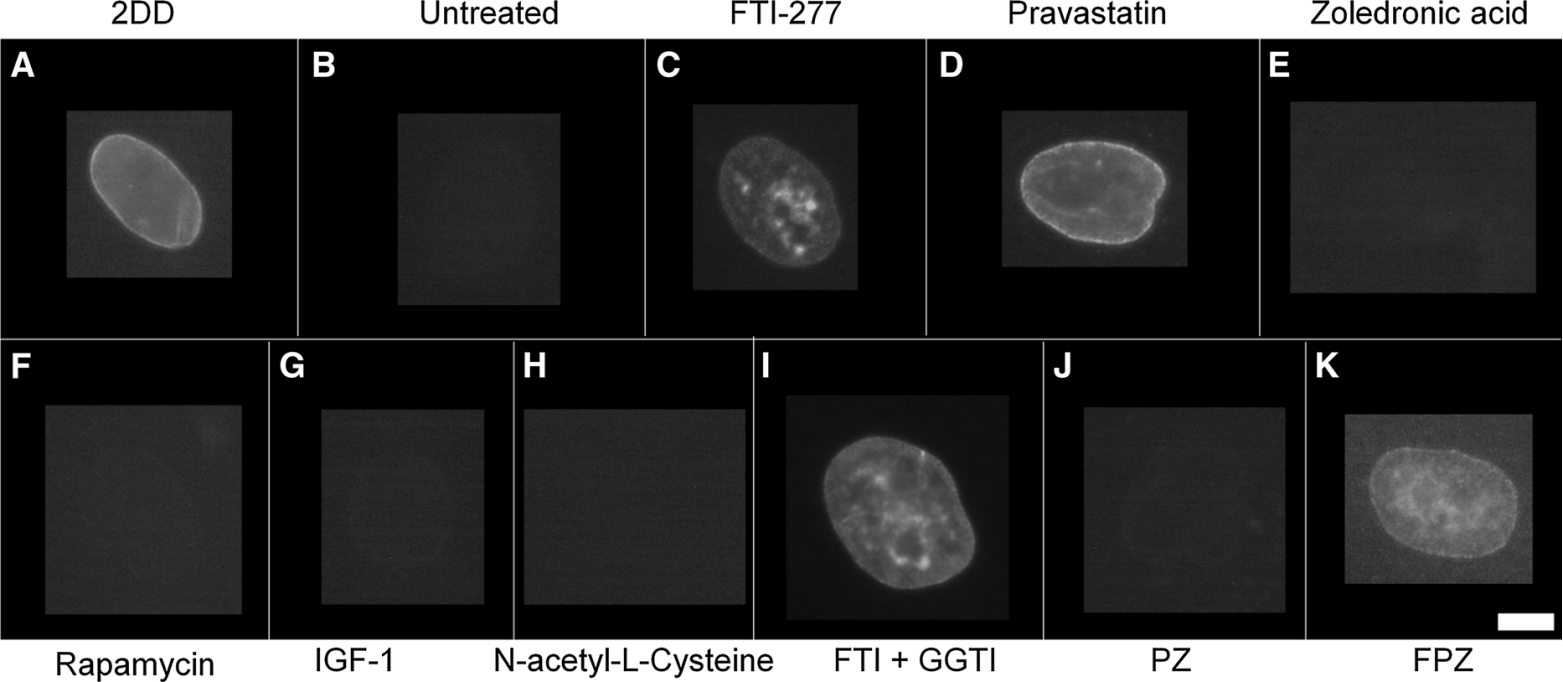
Representative images of lamin A staining of HGPS fibroblast nuclei. Representative images of HGPS nuclei fixed with methanol:acetone and stained using a lamin A specific primary antibody and a FITC-conjugated secondary antibody. Images captured at ×100 magnification using an Olympus BX-41 fluorescence microscope with an UPlanFl ×100/1.30 objective lens (Olympus Corporation, Japan). Images were captured with **a** Viewpoint GS digital camera and SmartCapture 3 software for Apple Mac (both Digital Scientific UK). The treatments are shown as a) untreated 2DD **b** untreated AG01972, **c** AG01972 with FTI-277 (2.5 µM for 24 h), **d** AG01972 with pravastatin (1 µM for 24 h), **e** AG01972 with zoledronic acid (1 µM for 24 h), **f** AG01972 with rapamycin (10 nM for 24 h), **g** AG01972 with insulin-like growth factor 1 (50.0 ng ml-1 for 24 h), **h** AG01972 with *N*-acetyl-l-cysteine (20 µM for 1 h), **i** AG01972 with FTI-277 and GGTI-2133 (both 2.5 µM for 24 h), **j** AG01972 with pravastatin and zoledronic acid (both 1 µM for 24 h) and **k** AG01972 with FTI-277 (2.5 µM for 24 h), pravastatin and zoledronic acid (both 1 µM for 24 h). Scale bar = 10 µM

**Fig. 4:**
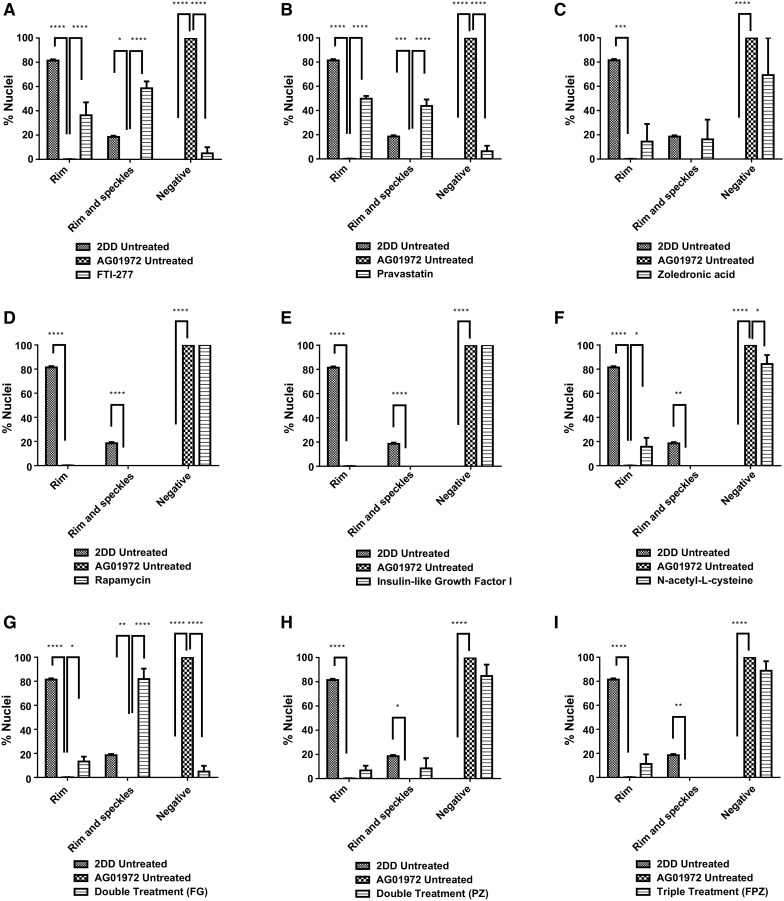
Scoring of lamin A stained AG01972 fibroblasts after drug treatment. 2DD and AG01972 nuclei fixed with methanol:acetone and stained using specific lamin A primary antibodies and FITC-conjugated secondary antibodies were scored by eye using an Olympus BX-41 fluorescence microscope with an UPlanFl ×100/1.30 objective lens (Olympus Corporation, Japan). The scoring patterns used for the analysis were rim, rim and speckles, speckles, and negative. Scoring results for the treatments are shown as **a** FTI-277 (2.5 µM for 24 h), **b** pravastatin (1 µM for 24 h), **c** zoledronic acid (1 µM for 24 h), **d** rapamycin (10 nM for 24 h), **e** insulin-like growth factor 1 (50.0 ng ml^−1^ for 24 h), **f***N*-acetyl-l-cysteine (20 µM for 1 h), **g** FTI-277 and GGTI-2133 (both 2.5 µM for 24 h), **h** pravastatin and zoledronic acid (both 1 µM for 24 h) and **i** FTI-277 (2.5 µM for 24 h), pravastatin and zoledronic acid (both 1 µM for 24 h). Error bars are SEM

In terms of lamin A distribution within nuclei (Figs. 3, 4), untreated HGPS fibroblasts exhibited a significant, substantial reduction in the proportion of cells where rim staining (0.2%) and rim and speckles staining was observed (0.0%) compared to fibroblast controls (81% and 19%, respectively). Untreated HGPS fibroblasts in this study predominantly exhibited negative staining for lamin A (94%) with some near negative, barely detectable staining (6%). Upon FTI-277 treatment (Fig. 4a), the fraction of HGPS cells with rim staining of mature lamin A (36%) and rim and speckles staining (59%) is significantly increased compared to untreated HGPS fibroblasts. The proportion of nuclei exhibiting negative staining (2%) is also significantly less than untreated HGPS fibroblasts (94%). Taken together, these data suggest that FTI rescues lamin A distribution in HGPS fibroblasts. Upon pravastatin treatment (Fig. 4b), the proportion of HGPS cells with rim staining (50%) and rim and speckles staining (44%) is also significantly increased compared to untreated HGPS fibroblasts. In addition, the proportion of nuclei exhibiting negative staining (2%) is also significantly less than untreated HGPS fibroblasts. Taken together, these data reveal that pravastatin also rescues lamin A distribution in HGPS fibroblasts. However, upon zoledronic acid treatment (Fig. 4c) there is a much less increase in the rim and rim and speckle staining fractions in addition to a large number of cells still showing no staining (67%), indicating that zoledronic acid does not significantly alter the lamin A distribution in HGPS fibroblasts. This is also seen in the HGPS cells treated with zoledronic acid in combination with FTI and pravastatin (Fig. 4h, i). In addition, rapamycin treatment (Fig. 4d) had no impact on the fraction of lamin A negative cells (94%). With IGF-1 treatment (Fig. 4e), again, there is a complete absence of both rim staining and rim and speckles staining, similar to untreated HGPS fibroblasts however, the proportion of nuclei exhibiting negative staining (70%) is still high but is significantly less than untreated HGPS fibroblasts (94%). Together, these data suggest that IGF1 seems to affect the lamin A distribution in HGPS fibroblasts, but does not constitute a full rescue of the protein to the nuclear envelope. Upon NAC treatment (Fig. 4f), the proportion of cells with rim staining (16%) is significantly increased compared to untreated HGPS fibroblasts and the proportion of nuclei exhibiting negative lamin A staining (68%) is significantly less than untreated HGPS fibroblasts but again is still high. These data reveal that NAC seems to have a positive affect the lamin A distribution in HGPS fibroblasts, but does not constitute a full rescue of the protein to the nuclear envelope.

Upon combination treatment of FTI-277 and GGTI-2133 (Fig. 4g), the proportion of cells with both rim staining (13%) and rim and speckles staining (82%) is significantly increased. The fraction of nuclei exhibiting negative staining (2%) is significantly less than HGPS fibroblasts. Upon combination treatment of pravastatin and zoledronic acid (Fig. 4h), both the rim staining (7%) and rim and speckles fractions (9%) are low, similar to untreated HGPS fibroblasts. However, the proportion of nuclei exhibiting negative staining (75%) is still high but significantly altered. Upon combination treatment of FTI-277, pravastatin and zoledronic acid (Fig. 4i), rim staining cells (11%) are significantly increased compared to HGPS fibroblasts and the proportion of HGPS cells. The proportion of nuclei exhibiting negative staining (67%) is significantly decreased compared to untreated HGPS fibroblasts (94%). In summary zoledronic acid seems to prevent a rescue of lamin A presence by FTI and pravastatin when used in combination.

### Lamin A/C distribution

**Fig. 5:**
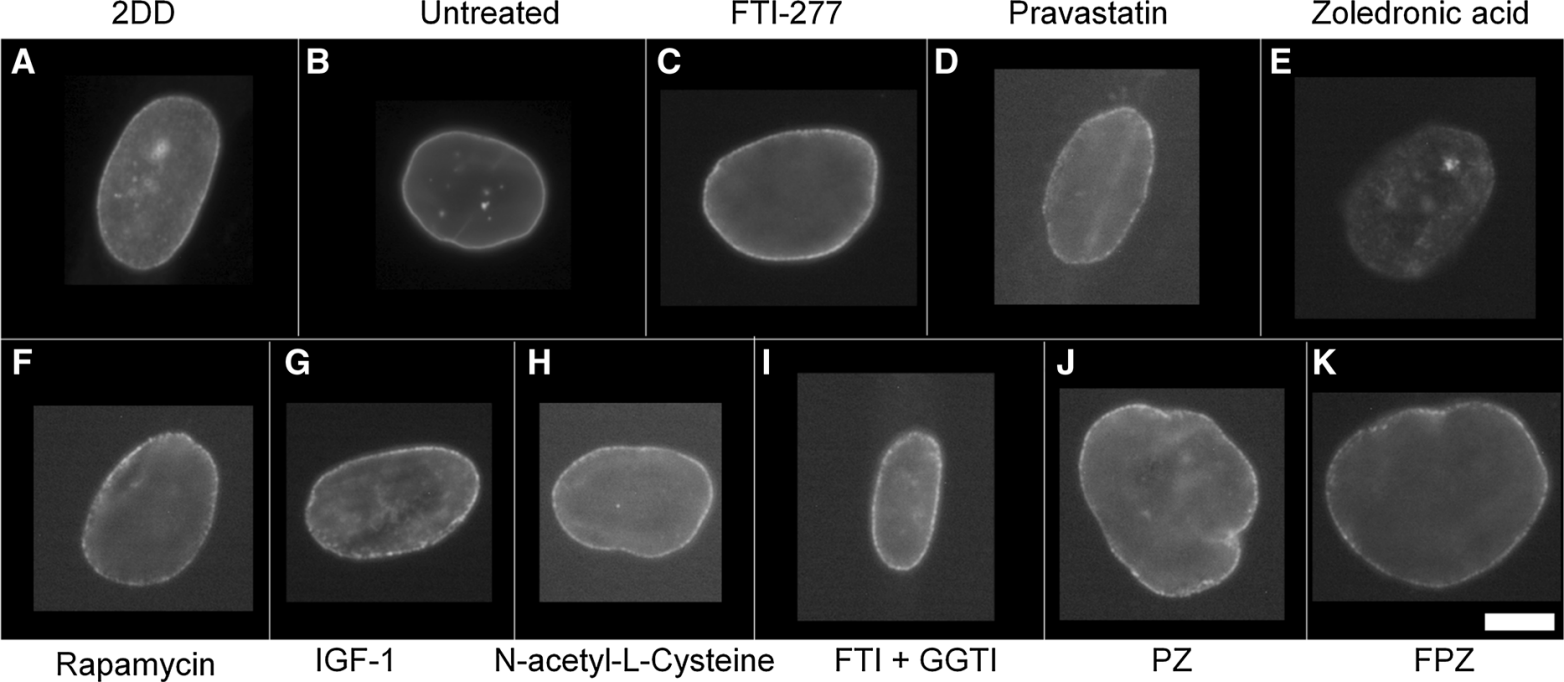
Representative images of lamin A/C staining of HGPS fibroblast nuclei. Representative images of HGPS nuclei fixed with methanol:acetone and stained using a lamin A/C primary antibody and a FITC-conjugated secondary antibody. Images captured at ×100 magnification using an Olympus BX-41 fluorescence microscope with an UPlanFl ×100/1.30 objective lens (Olympus Corporation, Japan). Images were captured with a Viewpoint GS digital camera and SmartCapture 3 software for Apple Mac (both Digital Scientific UK). The treatments are shown as **a** untreated 2DD **b** untreated AG01972, **c** AG01972 with FTI-277 (2.5 µM for 24 h), **d** AG01972 with pravastatin (1 µM for 24 h), **e** AG01972 with zoledronic acid (1 µM for 24 h), **f** AG01972 with rapamycin (10 nM for 24 h), **g** AG01972 with insulin-like growth factor 1 (50.0 ng ml-1 for 24 h), **h** AG01972 with *N*-acetyl-l-cysteine (20 µM for 1 h), **i** AG01972 with FTI-277 and GGTI-2133 (both 2.5 µM for 24 h), **j** AG01972 with pravastatin and zoledronic acid (both 1 µM for 24 h) and **k** AG01972 with FTI-277 (2.5 µM for 24 h), pravastatin and zoledronic acid (both 1 µM for 24 h). Scale bar = 10 µM

**Fig. 6:**
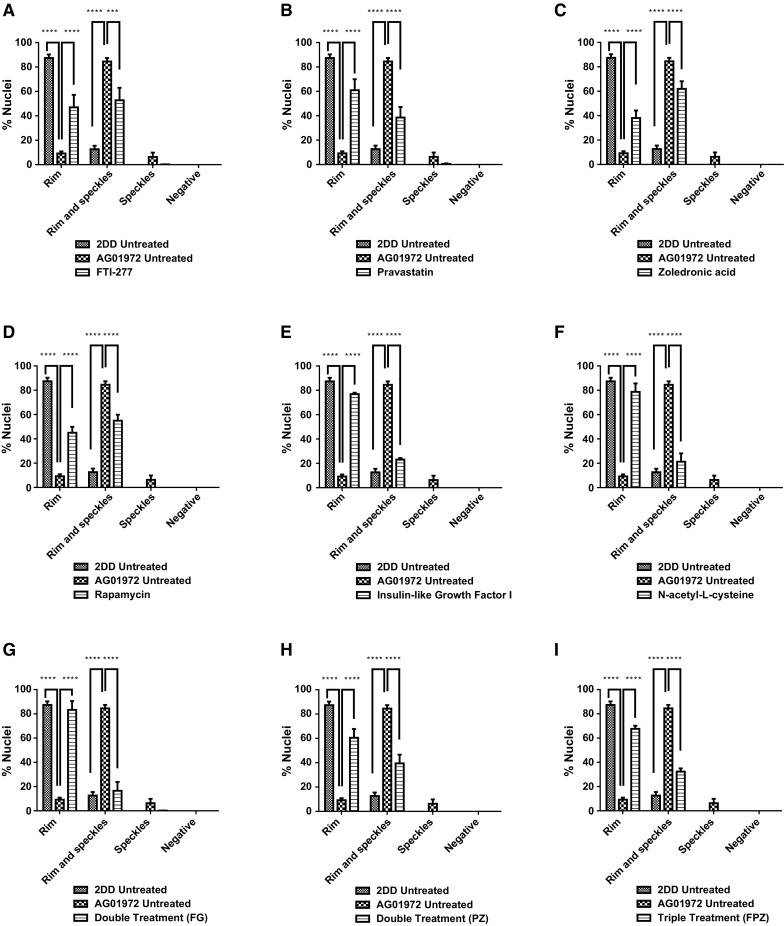
Scoring of lamin A/C stained AG01972 fibroblasts after drug treatment. 2DD and AG01972 nuclei fixed with methanol:acetone and stained using lamin A/C primary antibodies and FITC-conjugated secondary antibodies were scored by eye using an Olympus BX-41 fluorescence microscope with an UPlanFl ×100/1.30 objective lens (Olympus Corporation, Japan). The scoring patterns used for the analysis were rim, rim and speckles, speckles, and negative. Scoring results for the treatments are shown as **a** FTI-277 (2.5 µM for 24 h), **b** pravastatin (1 µM for 24 h), **c** zoledronic acid (1 µM for 24 h), **d** rapamycin (10 nM for 24 h), **e** insulin-like growth factor 1 (50.0 ng ml^−1^ for 24 h), f) *N*-acetyl-l-cysteine (20 µM for 1 h), **g** FTI-277 and GGTI-2133 (both 2.5 µM for 24 h), **h** pravastatin and zoledronic acid (both 1 µM for 24 h) and **i** FTI-277 (2.5 µM for 24 h), pravastatin and zoledronic acid (both 1 µM for 24 h). Error bars are SEM

In terms of anti-lamin A/C distribution within nuclei (Figs. 5, 6), untreated HGPS fibroblasts exhibited a significantly reduced proportion of rim only staining in cells (9%) compared to the control fibroblasts (87%). Furthermore, the untreated HGPS fibroblasts have a significantly higher fraction of cells that display internal speckles in addition to a rim stain for A-type lamins (85%) compared to control fibroblasts (13%). However, upon treatment with all the drugs and combinations (Fig. 6), the proportion of rim only staining for HGPS fibroblasts is significantly increased compared to untreated HGPS controls (38–83%) with IGF1, NAC and FTI + GGTI having the best effect and zoledronic acid the lesser effect. In addition, the proportion of HGPS cells with rim and speckle staining was significantly reduced (21–68%), again with zoledronic acid being the drug that affected the HGPS cells the least, implying that the presence of zoledronic acid is inhibiting rescue. The drug treatments that bring the HGPS cells closest to the control fibroblasts are IGF-1 (Fig. 6e), NAC (Fig. 6f), FTI + GGTI (Fig. 6g).

### Progerin distribution

**Fig. 7:**
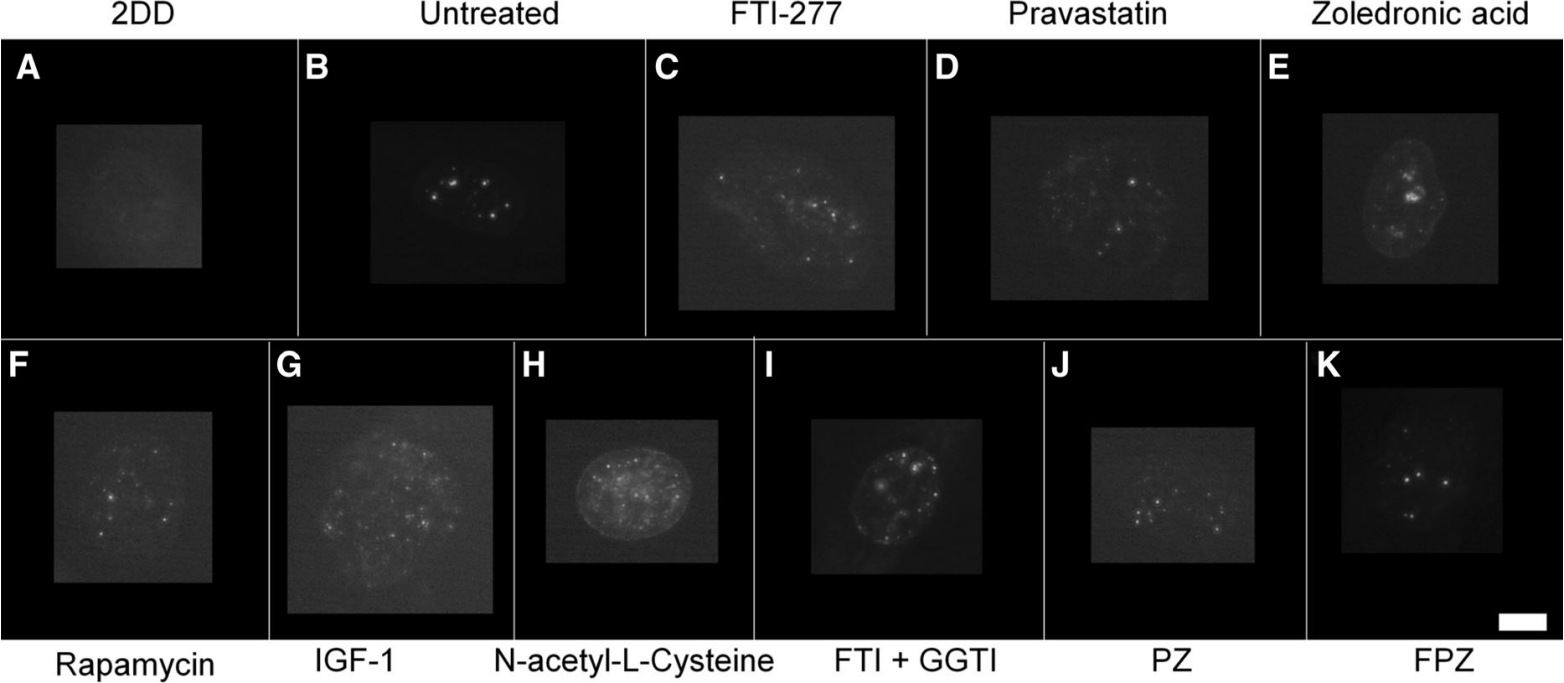
Representative images of progerin staining of HGPS fibroblast nuclei. HGPS nuclei fixed with methanol:acetone stained using a progerin primary antibody and a FITC-conjugated secondary antibody. Images captured at ×100 magnification using an Olympus BX-41 fluorescence microscope with an UPlanFl ×100/1.30 objective lens (Olympus Corporation, Japan). Images were captured with a Viewpoint GS digital camera and SmartCapture 3 software for Apple Mac (both Digital Scientific UK). The treatments are shown as **a** untreated 2DD **b** untreated AG01972, **c** AG01972 with FTI-277 (2.5 µM for 24 h), **d** AG01972 with pravastatin (1 µM for 24 h), **e** AG01972 with zoledronic acid (1 µM for 24 h), **f** AG01972 with rapamycin (10 nM for 24 h), **g** AG01972 with insulin-like growth factor 1 (50.0 ng ml-1 for 24 h), **h** AG01972 with *N*-acetyl-l-cysteine (20 µM for 1 h), **i** AG01972 with FTI-277 and GGTI-2133 (both 2.5 µM for 24 h), **j** AG01972 with pravastatin and zoledronic acid (both 1 µM for 24 h) and **k** AG01972 with FTI-277 (2.5 µM for 24 h), pravastatin and zoledronic acid (both 1 µM for 24 h). Scale bar = 10 µM

**Fig. 8:**
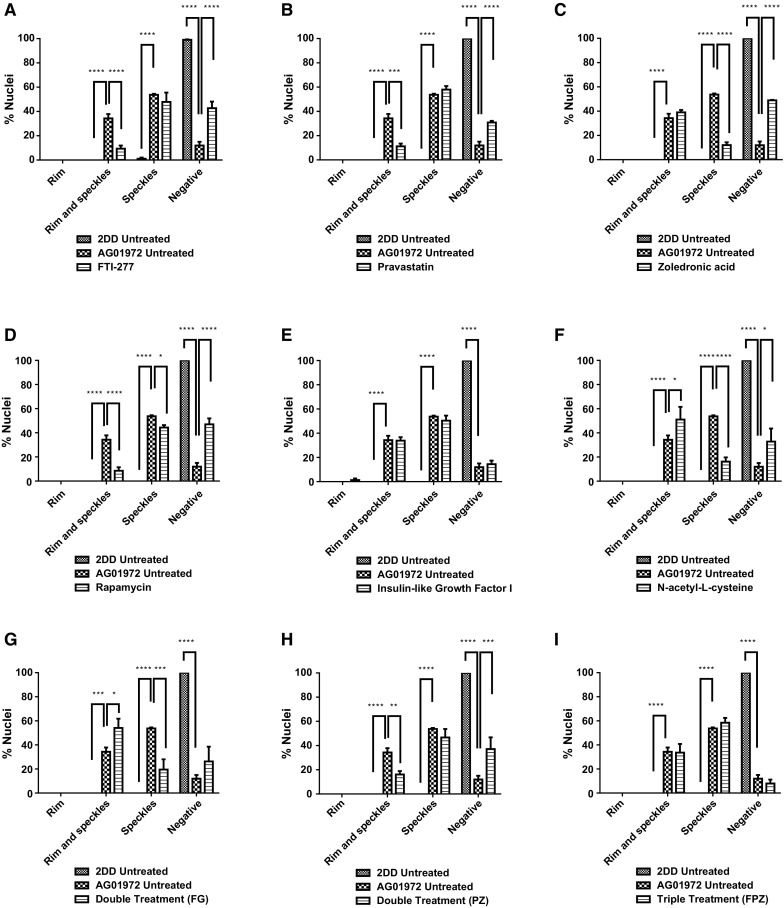
Scoring of progerin stained AG01972 fibroblasts after drug treatment. 2DD and AG01972 nuclei fixed with methanol:acetone and stained using progerin primary antibodies and FITC-conjugated secondary antibodies were scored by eye using an Olympus BX-41 fluorescence microscope with an UPlanFl ×100/1.30 objective lens (Olympus Corporation, Japan). The scoring patterns used for the analysis were rim, rim and speckles, speckles, and negative. Scoring results for the treatments are shown as **a** FTI-277 (2.5 µM for 24 h), **b** pravastatin (1 µM for 24 h), **c** zoledronic acid (1 µM for 24 h), **d** rapamycin (10 nM for 24 h), **e** insulin-like growth factor 1 (50.0 ng ml^−1^ for 24 h), **f***N*-acetyl-l-cysteine (20 µM for 1 h), **g** FTI-277 and GGTI-2133 (both 2.5 µM for 24 h), **h** pravastatin and zoledronic acid (both 1 µM for 24 h) and **i** FTI-277 (2.5 µM for 24 h), pravastatin and zoledronic acid (both 1 µM for 24 h). Error bars are SEM

Using an antibody specific for progerin (Fig. S1), the toxic truncated form of lamin A found in HGPS cells, reveals that most of the untreated HGPS fibroblasts exhibited progerin both at the rim with internal speckles but also 54% of cells with only internal accumulations of progerin. Interestingly, the control fibroblasts do not exhibit any staining of progerin at the nuclear edge but 1% of the cells contain progerin within internal speckles. These observations provide further evidence that progerin is produced by cells derived from healthy individuals (Figs. 7, 8). A number of the treatments do not reduce the fraction of cells with internal speckles of progerin i.e. FTI alone, pravastatin alone, rapamycin, IGF1, pravastatin and zoledronic acid in combination. However, the fraction of negative cells with respect to progerin staining is significantly increased from 12% for untreated HGPS cells for treatments with FTI (43%), pravastatin (31%), zoledronic acid (49%), rapamycin (47%), NAC (33%), pravastatin and zoledronic acid together (37%). This increase in negative cells seems to be due to the loss of progerin at the nuclear envelope since these staining fractions reduce with all these drug treatments, apart from zoledronic acid and NAC where the cells displaying only internal speckles are significantly reduced to 12 and 16%, respectively from 54%. IGF1 and combination treatment of FTI, pravastatin and zoledronic acid do not elicit any changes at all with respect to anti-progerin distribution. The drugs that work the best in this analysis are FTI and rapamycin creating the highest proportion of negative cells for HGPS cells.

### Lamin B1 and B2 distribution

**Fig. 9:**
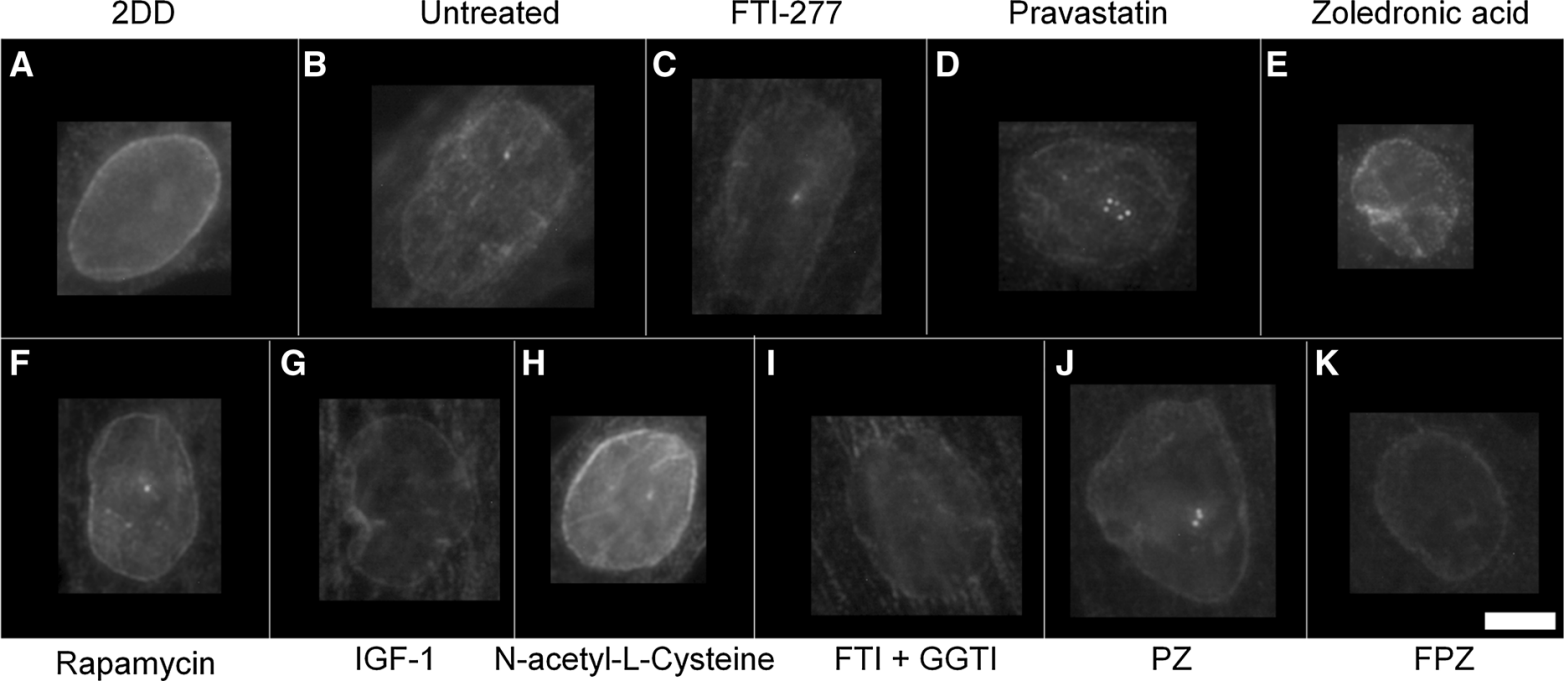
Representative images of Lamin B1 staining of HGPS fibroblast nuclei. HGPS nuclei fixed with methanol:acetone and stained using lamin B1 primary antibodies and FITC-conjugated secondary antibodies. Images captured at ×100 magnification using an Olympus BX-41 fluorescence microscope with an UPlanFl ×100/1.30 objective lens (Olympus Corporation, Japan). Images were captured with a Viewpoint GS digital camera and SmartCapture 3 software for Apple Mac (both Digital Scientific UK). The treatments are shown as **a** untreated 2DD **b** untreated AG01972, **c** AG01972 with FTI-277 (2.5 µM for 24 h), **d** AG01972 with pravastatin (1 µM for 24 h), **e** AG01972 with zoledronic acid (1 µM for 24 h), **f** AG01972 with rapamycin (10 nM for 24 h), **g** AG01972 with insulin-like growth factor 1 (50.0 ng ml-1 for 24 h), **h** AG01972 with *N*-acetyl-l-cysteine (20 µM for 1 h), **i** AG01972 with FTI-277 and GGTI-2133 (both 2.5 µM for 24 h), **j** AG01972 with pravastatin and zoledronic acid (both 1 µM for 24 h) and **k** AG01972 with FTI-277 (2.5 µM for 24 h), pravastatin and zoledronic acid (both 1 µM for 24 h). Scale bar = 10 µM

**Fig. 10:**
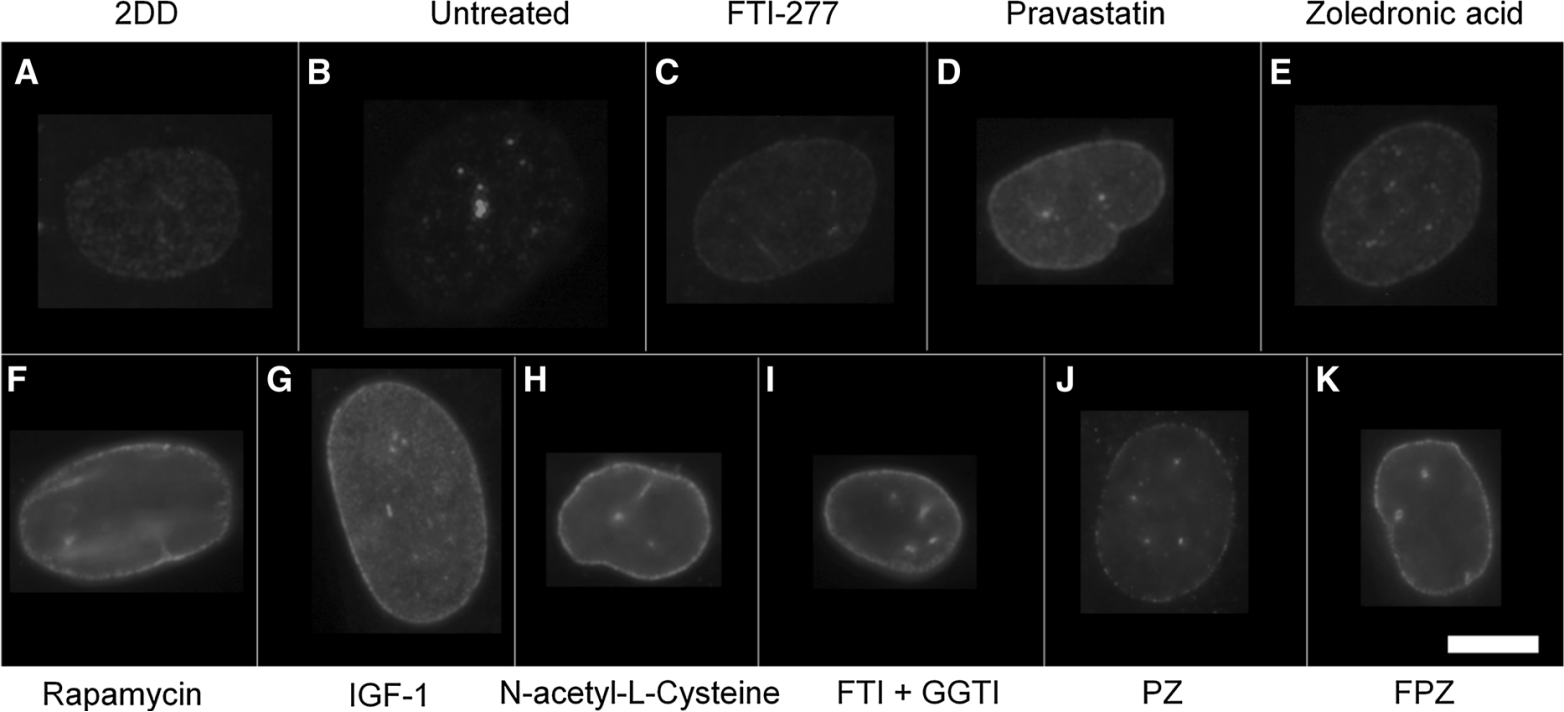
Representative images of Lamin B2 staining of HGPS fibroblast nuclei. HGPS nuclei fixed with methanol:acetone and stained using lamin B2 primary antibodies and FITC-conjugated secondary antibodies. Images captured at ×100 magnification using an Olympus BX-41 fluorescence microscope with an UPlanFl ×100/1.30 objective lens (Olympus Corporation, Japan). Images were captured with a Viewpoint GS digital camera and SmartCapture 3 software for Apple Mac (both Digital Scientific UK). The treatments are shown as **a** untreated 2DD **b** untreated AG01972, **c** AG01972 with FTI-277 (2.5 µM for 24 h), **d** AG01972 with pravastatin (1 µM for 24 h), **e** AG01972 with zoledronic acid (1 µM for 24 h), **f** AG01972 with rapamycin (10 nM for 24 h), **g** AG01972 with insulin-like growth factor 1 (50.0 ng ml-1 for 24 h), **h** AG01972 with *N*-acetyl-L-cysteine (20 µM for 1 h), **i** AG01972 with FTI-277 and GGTI-2133 (both 2.5 µM for 24 h), **j** AG01972 with pravastatin and zoledronic acid (both 1 µM for 24 h) and **k** AG01972 with FTI-277 (2.5 µM for 24 h), pravastatin and zoledronic acid (both 1 µM for 24 h). Scale bar = 10 µM. Scale bar = 10 µM

**Fig. 11:**
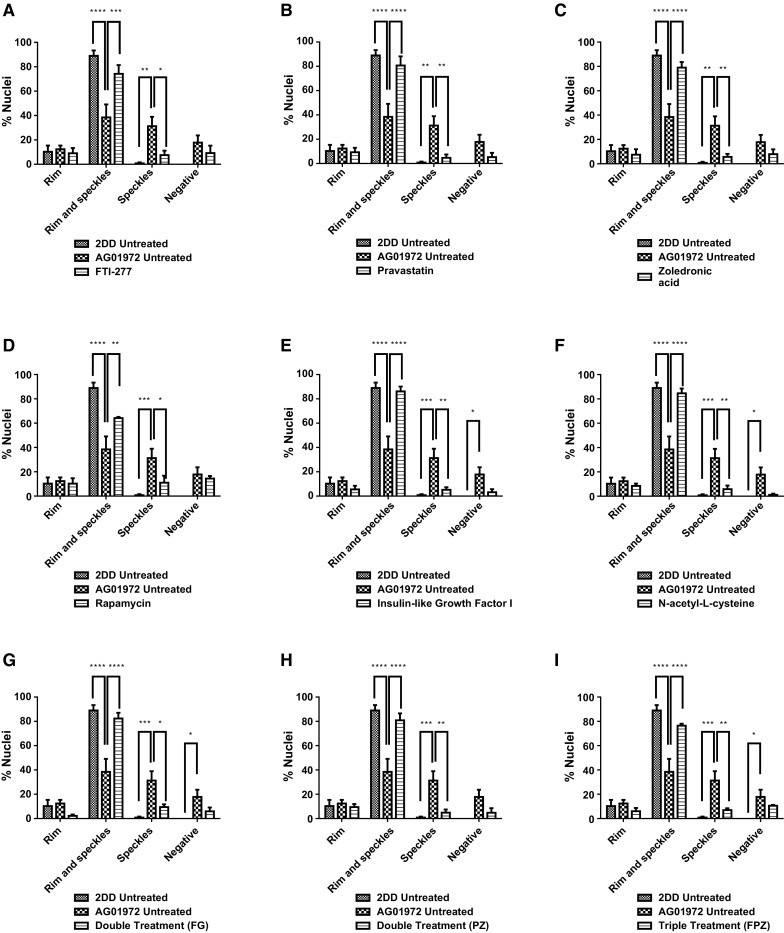
Scoring of Lamin B2 stained AG01972 fibroblasts after drug treatment. 2DD and AG01972 nuclei fixed with methanol:acetone and stained using lamin B2 primary antibodies and FITC-conjugated secondary antibodies were scored by eye using an Olympus BX-41 fluorescence microscope with an UPlanFl ×100/1.30 objective lens (Olympus Corporation, Japan). The scoring patterns used for the analysis were rim, rim and speckles, speckles, and negative. Scoring results for the treatments are shown as **a** FTI-277 (2.5 µM for 24 h), **b** pravastatin (1 µM for 24 h), **c** zoledronic acid (1 µM for 24 h), **d** rapamycin (10 nM for 24 h), **e** insulin-like growth factor 1 (50.0 ng ml^−1^ for 24 h), **f***N*-acetyl-L-cysteine (20 µM for 1 h), **g** FTI-277 and GGTI-2133 (both 2.5 µM for 24 h), **h** pravastatin and zoledronic acid (both 1 µM for 24 h) and i) FTI-277 (2.5 µM for 24 h), pravastatin and zoledronic acid (both 1 µM for 24 h). Error bars are SEM

To determine the impact of our outlined drug treatments on lamin B1 and B2 distribution, we repeated the analysis of treated and untreated HGPS fibroblasts following indirect immunofluorescence with specific antibodies to the two B-type lamins. No significant changes in lamin B1 distribution was observed for any of the conditions outlined within our study (Fig. 9), indicating an independence of lamin B1. The majority of control fibroblasts have a definite rim stain of lamin B2 with some internal speckles (89%) (Fig. 10). Untreated HGPS fibroblasts on the other hand exhibit significantly lower fraction of cells with rim and speckles staining (39%). In addition, untreated HGPS fibroblasts exhibit significantly higher proportion of speckles only staining (31%) and as well as negative staining (18%). All the drug treatments improved the HGPS cells with respect to lamin B2 by exhibiting a reduction in the fraction of lamin B2 speckle-only HGPS cells and increasing the fraction of cells with both a lamin B2 rim and internal foci, resembling the distribution observed in control fibroblasts (Fig. 11).

## Discussion

The goal of this investigation was to assess the effects of different drugs on nuclear lamin distribution in HGPS fibroblasts, with the aim to reveal drug(s) or combinations of drugs that could generate distribution patterns within HGPS cells that most resemble patterns within normal control fibroblasts. Fibroblasts were grown in the presence of drugs and combinations of drugs that represent regimes being used within the clinic to treat HGPS. Furthermore, drugs that have been suggested as potential treatments were also investigated. The efficacy of proposed drug treatments for HGPS, up until recently, focused on nuclear shape since drugs in development for HGPS have been demonstrated to improve the morphology of the misshapen HGPS (Toth et al. 2005, Wang et al. 2010, Cao et al. 2011). This is a simple assay and does not inform entirely about other issues within nuclei that are affected by the presence of progerin, such as genome behaviour (Goldman et al. 2002, 2004; Meaburn et al. 2005, 2007; Mehta et al. 2011; Bikkul et al. 2018), DNA repair and replication (Richards et al. 2011; Bikkul et al. 2018) and the presence and distribution of nuclear structures (Mehta et al. 2010). More importantly, many HGPS cells in culture, especially at early passage, do not have misshapen nuclei. They have normal ellipsoid shape nuclei (Bridger and Kill 2004; Mehta et al. 2010). This study assesses the presence and distribution of six nuclear lamin proteins using nine different drug regimes.

FTIs, statins and bisphosphonates are intended to target progerin toxicity by preventing farnesylation through the mevalonate pathway and thus prevent its accumulation in the nuclear membrane. However, farnesylation is an important, normal biological process that modifies a number of proteins, including prelamin A. In this study we found that FTI, pravastatin and zoledronic acid did indeed bring about a significant increase in the fraction of cells negative for progerin, with FTI, pravastatin and zoledronic acid, in combination, most effective. However, we also observed cells that had progerin within the nucleoplasm in speckles and that the fraction of these cells did not change with FTI and pravastatin. This could imply that there are two populations of progerin, one at the nuclear envelope and one in the nuclear interior. At present, it is not possible to comment whether one population is more deleterious to the cell than the other; since progerin at the nuclear envelope probably causes mitotic catastrophe and create issues with chromatin organisation; whereas the internal population may affect transcription, DNA replication and repair (Bridger and Kill 2004) and nucleolar structure (Mehta et al. 2010). Two drugs that worked well for HGPS and reduced the fraction of cells displaying both a progerin rim and internal foci were zoledronic acid and NAC, with zoledronic acid superior to NAC with respect to the number of progerin negative cells it generated. What was interesting in our study with respect to progerin staining was that the combinations of drugs did not perform better than their solo counterparts, indeed sometimes worse. It should be noted however that FTI + GGTI did reduce the fraction of cells displaying progerin speckles only. FTI and pravastatin also performed well separately by restoring normal lamin A staining to the HGPS cultures which were mostly negative before treatment. NAC and FTI + GGTI again also restored normal lamin A staining. This could explain why chromosome territory positions are restored in HGPS cells particularly treated with these drugs (Bikkul et al. 2018), since they restore a more normal lamin A distribution and presumably allow lamina associated domains (LADs) to be tethered at the nuclear envelope correctly. However, a note of caution is required with these drugs that prevent the farnesylation of progerin also lead to accumulations of prelamin A at the nuclear envelope (as also shown by Adam et al. 2013) and in the nucleoplasm as speckles. Indeed, all these drugs singularly, and in combination, led to rim and internal speckle distributions—with strong rim staining seen for treatments with FTI, zoledronic acid and all the combinations (FTI + GGTI, PZ, FPZ). Our study extends this by showing that other inhibitors of other signalling proteins in the mevalonate pathway, such as pravastatin and zoledronic acid, also lead to cells exhibiting accumulation of prelamin A at the nuclear rim, but to a lesser extent. Importantly, prelamin A is known to be a toxic protein if left uncleaved and embedded in the nuclear rim (Fong et al. 2006; Dominici et al. 2009; Davies et al. 2011). Pravastatin performed the best of the drugs affecting farnesylation, since it only produced a weak rim of prelamin A staining. Assessing this study and our other recent study (Bikkul et al. 2018) in combination it seems that the presence of prelamin A at the nuclear envelope does not prevent LAD binding, since gene-poor chromosomes are located to the nuclear envelope after drug treatments.

Much is being made of rapamycin as a new treatment for HGPS and even normal ageing (Mendelsohn and Larrick 2012). In this study rapamycin treatment does increase the proportion of progerin negative cells but does not alter the fraction of cells with internal progerin speckles. We also saw a reduction in prelamin A with rapamycin probably due to the activation of autophagic pathways and degradation of prelamin A that have been previously been reported upon treatment with rapamycin (Cao et al. 2011). A drug that affects both the progerin populations would seem to be a more sensible choice, that did not have adverse effects elsewhere—that drug could be NAC with respect to this study since it has a positive effect on both progerin populations and does not have deleterious effects on other lamin distributions such as accumulation of prelamin A; whereas zoledronic acid does reduce both progerin pools but does not restore normal lamin A staining.

HGPS cells also display abnormal distributions of lamin B2. Lamin B2 was observed located away from the nuclear envelope as speckles within the nucleoplasm. This implies that lamin B2 requires lamin A to be localised at the nuclear envelope or that its journey is impeded by progerin. As opposed to lamin A/C and lamin B1, very few studies have been conducted studying lamin B2 localisation following drug treatments in HGPS fibroblasts. However, the study by Adam et al. (2013) saw that there was an increase in lamin B2 speckles due to unfarnesylated lamin B2 accumulating in the nucleoplasm. The results obtained in our study however suggest that untreated HGPS fibroblasts already have a high proportion of lamin B2 speckles in the nucleoplasm compared to control fibroblasts, and that drug treatments were able to increase rim staining whilst at the same time, reduce internal speckles, bringing the HGPS cells towards that of control cells.

Wild-type fibroblasts used in this study do not exhibit prelamin A at the nuclear rim. In this study regulators of the mTOR pathway, such as rapamycin and IGF-1 do not induce HGPS cells to exhibit prelamin A accumulation at the nuclear membrane. There is a significant reduction in nuclear speckles, with no accumulation of prelamin A at the nuclear rim. These data indicate that drugs that are able to manipulate the mTOR pathway may be beneficial in terms of treatment of HGPS. Furthermore, we also revealed that suggested potential drug treatments IGF1 and NAC also have a positive effect on HGPS cells without producing a concentration of prelamin A at the nuclear rim.

The specific lamin A antibody used during this study targets residues 598–611 at the C terminus of lamin A. In this study the antibody exhibits very weak or negative staining in untreated A01972 fibroblasts, indicating that very little full length lamin A protein is actually produced in these cells. The fact that HGPS fibroblasts show a large proportion of negative staining is reflected in previous studies that have used the same specific lamin A antibody to immunostain HGPS fibroblast cell lines (Bridger and Kill 2004). Instead of producing lamin A, AG01972 produces progerin, which forms aggregates in the nucleoplasm as well as accumulating at the nuclear envelope. The HGPS cells must also produce lamin C since the anti-lamin A/C staining is evident in these cells at the nuclear rim. Drug treatments such as FTI elevate the amount of lamin A at the nuclear envelope, and at the same time lead to a reduction of progerin at the nuclear envelope. However, the blocking of farnesylation by FTI treatments does not lead to a clearance of progerin protein from the nucleoplasm. This relocation of progerin in response to FTI treatment is in strong agreement with previous work carried out using in vitro mouse models (Yang et al. 2005). In addition, our study shows that pravastatin, another inhibitor of the mevalonate pathway, can bring about similar effects as FTI treatment in human cells.

The drugs may redistribute lamin A/C from nucleoplasmic speckles to the nuclear membrane, reduce nucleoplasmic speckles themselves or reduce their association with lamin A/C. It is known that progerin forms insoluble aggregates in the nucleoplasm (Cao et al. 2011). It has previously been described that blocking farnesylation of lamin A can lead to the redistribution of the protein away from the inner nuclear membrane (Wang et al. 2012), which may explain why FTI-277, pravastatin and zoledronic acid did not completely rescue nucleoplasmic foci back to control levels. Drugs that do not manipulate farnesylation performed better.

In conclusion, the results of this study have potentially far-reaching implications for the treatment of sufferers with HGPS since the drugs being used in clinical trial are a FTI—lonafarnib, and a rapalogue—everolimus.

## Electronic supplementary material

Below is the link to the electronic supplementary material.

Supplementary material 1 (DOCX 103 kb)

## Abbreviations

DSB: Double stand break
FTI: Farnesyltransferase inhibitors
GGTI: Geranylgeranyltransferase inhibitor
GH: Growth hormone
HGPS: Hutchinson–Gilford progeria syndrome
ICMT: Isoprenyl-cysteine-carboxy-methyltransferase
IGF-1: Insulin-like growth factor 1
mTOR: Mammalian target of rapamycin
ROS: Reactive oxygen species
