# Supplementary material for: Presence and distribution of progerin in HGPS cells is ameliorated by drugs that impact on the mevalonate and mTOR pathways
Source: Biogerontology. 2019 Apr 30;20(3):337–58. doi: 10.1007/s10522-019-09807-4 (PMC6535420; doi:10.1007/s10522-019-09807-4)
Supplement: Supplementary file 1 — Supplementary material 1 (DOCX 103 kb) [file 10522_2019_9807_MOESM1_ESM.docx]

Supplementary Tables

Supp Table 1: Prelamin A

|  | Nuclei exhibiting scoring pattern (%± SEM, n=3) | | | |
| --- | --- | --- | --- | --- |
| Drug(s) | Negative | Speckles | Weak rim + Speckles | Strong rim + Speckles |
| 2DD Control | 100.0 ± 0.0 | 0.0 ± 0.0 | 0.0 ± 0.0 | 0.0 ± 0.0 |
| AG01972 Control | 23.4 ± 3.6 | 54.6 ± 9.2 | 20.2 ± 7.6 | 1.8 ± 1.4 |
| FTI-277 | 2.7 ± 1.8 | 3.5 ± 3.3 | 11.5 ± 3.2 | 82.3 ± 5.3 |
| Pravastatin | 22.6 ± 15.4 | 15.8 ± 6.0 | 57.8 ± 12.4 | 3.8 ± 3.8 |
| Zoledronic acid | 8.9 ± 3.6 | 15.2 ± 2.1 | 53.8 ± 8.6 | 22.1 ± 14.2 |
| Rapamycin | 70.9 ± 14.3 | 25.2 ± 10.5 | 3.8 ± 3.8 | 0.1 ± 0.1 |
| IGF-1 | 55.0 ± 19.4 | 23.6 ± 13.4 | 19.2 ± 8.6 | 2.3 ± 1.1 |
| N-acetyl-L-Cysteine | 54.9 ± 22.2 | 29.7 ± 15.5 | 14.6 ± 6.6 | 0.8 ± 0.6 |
| FG | 10.6 ± 5.0 | 1.4 ± 0.6 | 19.1 ± 8.3 | 68.9 ± 6.8 |
| PZ | 23.3 ± 12.4 | 16.6 ± 6.2 | 59.3 ± 11.6 | 0.8 ± 0.4 |
| FPZ | 2.9 ± 2.0 | 0.1 ± 0.1 | 12.2 ± 1.9 | 84.8 ± 3.5 |

Supp Table 2 - Lamin A

|  | Nuclei exhibiting scoring pattern (%± SEM, n=3) | | |
| --- | --- | --- | --- |
| Drug(s) | Rim | Rim and Speckles | Negative |
| 2DD Control | 81.4 ± 1.0 | 18.6 ± 1.0 | 0.0 ± 0.0 |
| AG01972 Control | 0.2 ± 0.2 | 0.0 ± 0.0 | 99.8 ± 0.2 |
| FTI-277 | 36.3 ± 10.7 | 58.6 ± 5.7 | 5.0 ± 5.0 |
| Pravastatin | 49.8 ± 2.2 | 43.8 ± 5.4 | 6.4 ± 4.5 |
| Zoledronic acid | 14.5 ± 14.5 | 16.3 ± 16.3 | 69.3 ± 30.7 |
| Rapamycin | 0.0 ± 0.0 | 0.0 ± 0.0 | 100.0 ± 0.0 |
| IGF-1 | 0.0 ± 0.0 | 0.0 ± 0.0 | 100.0 ± 0.0 |
| N-acetyl-L-Cysteine | 15.7 ± 7.4 | 0.0 ± 0.0 | 84.3 ± 7.4 |
| FG | 13.3 ± 3.9 | 81.9 ± 8.7 | 4.8 ± 4.8 |
| PZ | 6.8 ± 3.9 | 8.4 ± 8.4 | 84.8 ± 9.4 |
| FPZ | 11.2 ± 8.0 | 0.0 ± 0.0 | 88.8 ± 8.0 |

Supp Table 3 - Lamin A/C

|  | Nuclei exhibiting scoring pattern (%± SEM, n=3) | | | |
| --- | --- | --- | --- | --- |
| Drug(s) | Rim | Rim and Speckles | Speckles | Negative |
| 2DD Control | 87.4 ± 2.9 | 12.6 ± 2.9 | 0.0 ± 0.0 | 0.0 ± 0.0 |
| AG01972 Control | 9.2 ± 1.7 | 84.6 ± 2.8 | 6.3 ± 3.6 | 0.0 ± 0.0 |
| FTI-277 | 47.0 ± 10.3 | 52.8 ± 10.2 | 0.2 ± 0.1 | 0.0 ± 0.0 |
| Pravastatin | 60.9 ± 9.0 | 38.5 ± 8.7 | 0.5 ± 0.4 | 0.0 ± 0.0 |
| Zoledronic acid | 38.0 ± 6.2 | 62.0 ± 6.2 | 0.0 ± 0.0 | 0.0 ± 0.0 |
| Rapamycin | 45.0 ± 4.9 | 55.0 ± 4.9 | 0.0 ± 0.0 | 0.0 ± 0.0 |
| IGF-1 | 76.9 ± 1.2 | 23.1 ± 1.2 | 0.0 ± 0.0 | 0.0 ± 0.0 |
| N-acetyl-L-Cysteine | 78.7 ± 6.9 | 21.3 ± 6.9 | 0.0 ± 0.0 | 0.0 ± 0.0 |
| FG | 83.3 ± 7.2 | 16.5 ± 7.3 | 0.2 ± 0.2 | 0.0 ± 0.0 |
| PZ | 60.6 ± 7.2 | 39.4 ± 7.2 | 0.0 ± 0.0 | 0.0 ± 0.0 |
| FPZ | 67.6 ± 2.6 | 32.4 ± 2.6 | 0.0 ± 0.0 | 0.0 ± 0.0 |

Supp Table 4 - Progerin

|  | Nuclei exhibiting scoring pattern (%± SEM, n=3) | | | |
| --- | --- | --- | --- | --- |
| Drug(s) | Rim | Rim and Speckles | Speckles | Negative |
| 2DD Control | 0.0 ± 0.0 | 0.0 ± 0.0 | 1.0 ± 1.0 | 99.0 ± 1.0 |
| AG01972 Control | 0.0 ± 0.0 | 34.3 ± 3.6 | 53.7 ± 0.8 | 12.0 ± 3.1 |
| FTI-277 | 0.0 ± 0.0 | 9.4 ± 2.6 | 47.9 ± 7.7 | 42.7 ± 5.4 |
| Pravastatin | 0.0 ± 0.0 | 11.2 ± 2.3 | 58.0 ± 2.9 | 30.8 ± 1.4 |
| Zoledronic acid | 0.0 ± 0.0 | 39.0 ± 2.0 | 12.0 ± 2.3 | 48.9 ± 0.4 |
| Rapamycin | 0.0 ± 0.0 | 8.6 ± 2.9 | 44.3 ± 2.0 | 47.1 ± 4.9 |
| IGF-1 | 1.3 ± 1.3 | 33.9 ± 2.8 | 50.4 ± 4.2 | 14.5 ± 2.9 |
| N-acetyl-L-Cysteine | 0.0 ± 0.0 | 51.0 ± 10.6 | 16.2 ± 3.6 | 32.8 ± 10.8 |
| FG | 0.0 ± 0.0 | 54.2 ± 7.6 | 19.5 ± 8.5 | 26.3 ± 12.2 |
| PZ | 0.0 ± 0.0 | 16.2 ± 2.8 | 46.7 ± 6.9 | 37.1 ± 9.7 |
| FPZ | 0.0 ± 0.0 | 33.6 ± 7.2 | 58.4 ± 4.0 | 8.0 ± 3.3 |

Supp Table 5 - Lamin B2

|  | Nuclei exhibiting scoring pattern (%± SEM, n=3) | | | |
| --- | --- | --- | --- | --- |
| Drug(s) | Rim | Rim and Speckles | Speckles | Negative |
| 2DD Control | 10.3 ± 5.1 | 88.9 ± 4.4 | 0.8 ± 0.8 | 0.0 ± 0.0 |
| AG01972 Control | 12.4 ± 3.0 | 38.5 ± 10.7 | 31.3 ± 7.7 | 17.8 ± 5.9 |
| FTI-277 | 9.1 ± 4.3 | 74.2 ± 7.2 | 7.5 ± 3.7 | 9.2 ± 6.1 |
| Pravastatin | 9.3 ± 3.7 | 80.7 ± 7.5 | 4.7 ± 2.7 | 5.3 ± 3.6 |
| Zoledronic acid | 7.5 ± 4.5 | 79.0 ± 4.6 | 5.6 ± 2.5 | 7.9 ± 4.0 |
| Rapamycin | 10.3 ± 4.5 | 64.2 ± 1.0 | 11.0 ± 5.8 | 14.5 ± 2.1 |
| IGF-1 | 5.5 ± 3.1 | 86.1 ± 4.0 | 5.2 ± 2.1 | 3.2 ± 2.5 |
| N-acetyl-L-Cysteine | 8.5 ± 1.9 | 84.6 ± 3.9 | 5.8 ± 3.0 | 1.2 ± 1.0 |
| FG | 2.2 ± 1.1 | 82.4 ± 4.7 | 9.4 ± 2.3 | 6.0 ± 3.2 |
| PZ | 9.5 ± 2.5 | 80.9 ± 5.7 | 4.8 ± 2.6 | 4.8 ± 3.7 |
| FPZ | 6.0 ± 2.7 | 76.5 ± 1.5 | 6.9 ± 1.5 | 10.6 ± 0.7 |

Supplementary Figure 1

**Western blot with anti-Progerin Antibody.**


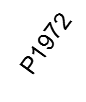

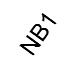

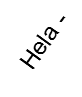

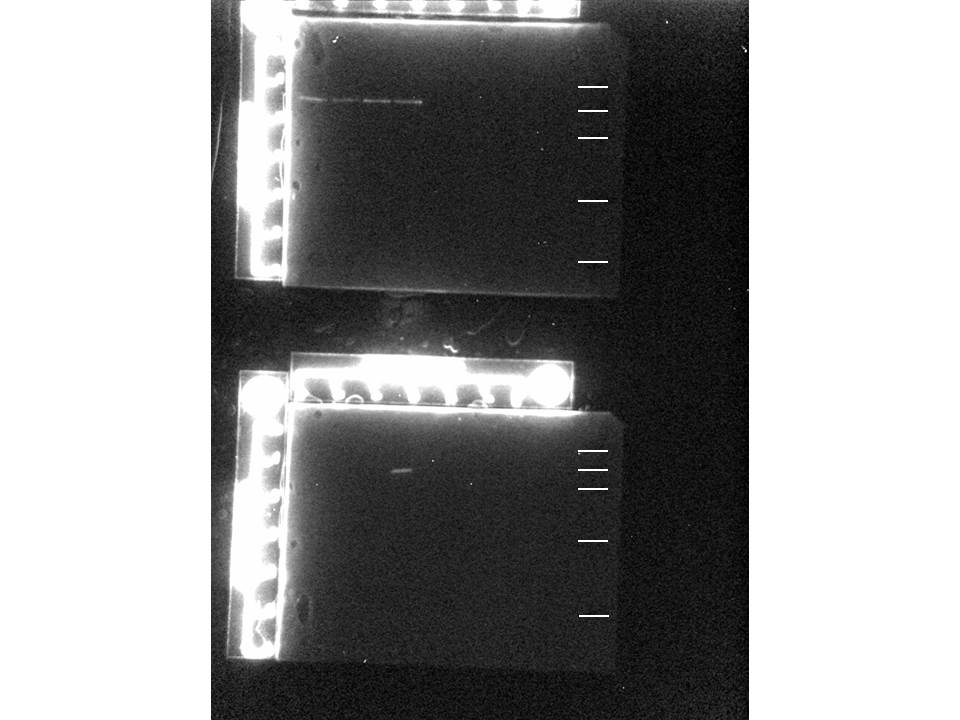


**Anti-Progerin Western Blot**

Hela cells, control fibroblasts (NB1) and AG01972 HGPS fibroblasts (2x105 cells per lane) were loaded onto a 10% SDS-PAGE gel. A western blot was performed with anti-Progerin mAb 13A4 (Enzo Life Sciences) diluted 1:1000 in PBS/1% dried milk. The secondary antibody was anti-mouse IgG, HRP-linked, diluted in 1:20.000. The signal was detected through Enhanced Chemiluminescence (ECL-ChemiDoc MP system - BioRad©). Markers were 100 kDa, 60kDa, 45kDa, 20kDa and 8kDa.
